# Corneal Deposits Following Combined Gatifloxacin and Prednisolone Therapy Post-Keratoplasty: A Report of a Rare Case

**DOI:** 10.7759/cureus.64283

**Published:** 2024-07-10

**Authors:** Chaitanya Modgil, Pranav More, Abhay Lune, Iqra Mushtaq

**Affiliations:** 1 Ophthalmology, Dr. D. Y. Patil Medical College, Hospital and Research Centre, Pune, IND; 2 Ophthalmology (Cornea), Dr. D. Y. Patil Medical College, Hospital and Research Centre, Pune, IND

**Keywords:** corneal scrapping, keratoplasty, prednisolone, gatifloxacin, corneal deposit

## Abstract

Corneal deposits associated with topical medications, particularly fluoroquinolones, are a recognized complication in ophthalmic practice. We present a case of a 66-year-old female with pseudophakic bullous keratopathy who developed corneal crystalline deposits following prolonged use of gatifloxacin and prednisolone eye drops post-penetrating keratoplasty. The patient presented with diminished vision and significant corneal opacity in the affected eye. Anterior segment examination and OCT imaging confirmed deposits extending from the epithelium to the anterior stroma. Management included corneal scrapping and transition to topical tobramycin and propylene glycol eye drops, resulting in the resolution of deposits and improvement in vision. This case underscores the importance of vigilant monitoring and judicious use of topical medications to mitigate adverse effects in high-risk ophthalmic patients undergoing corneal procedures.

## Introduction

Corneal deposits have been documented with various topical medications, including fluoroquinolones. Fluoroquinolones are a class of bactericidal antibiotics that exert their action by inhibiting DNA gyrase (topoisomerase II) and topoisomerase IV in bacteria, thereby disrupting bacterial genetic replication [1]. Corneal deposits have been observed following topical administration of several fluoroquinolones, such as ciprofloxacin, ofloxacin, gatifloxacin, sparfloxacin, moxifloxacin and tosufloxacin [2]. The present case study reported gatifloxacin- and prednisolone-induced corneal deposits post-keratoplasty.

## Case presentation

**Figure 1:**
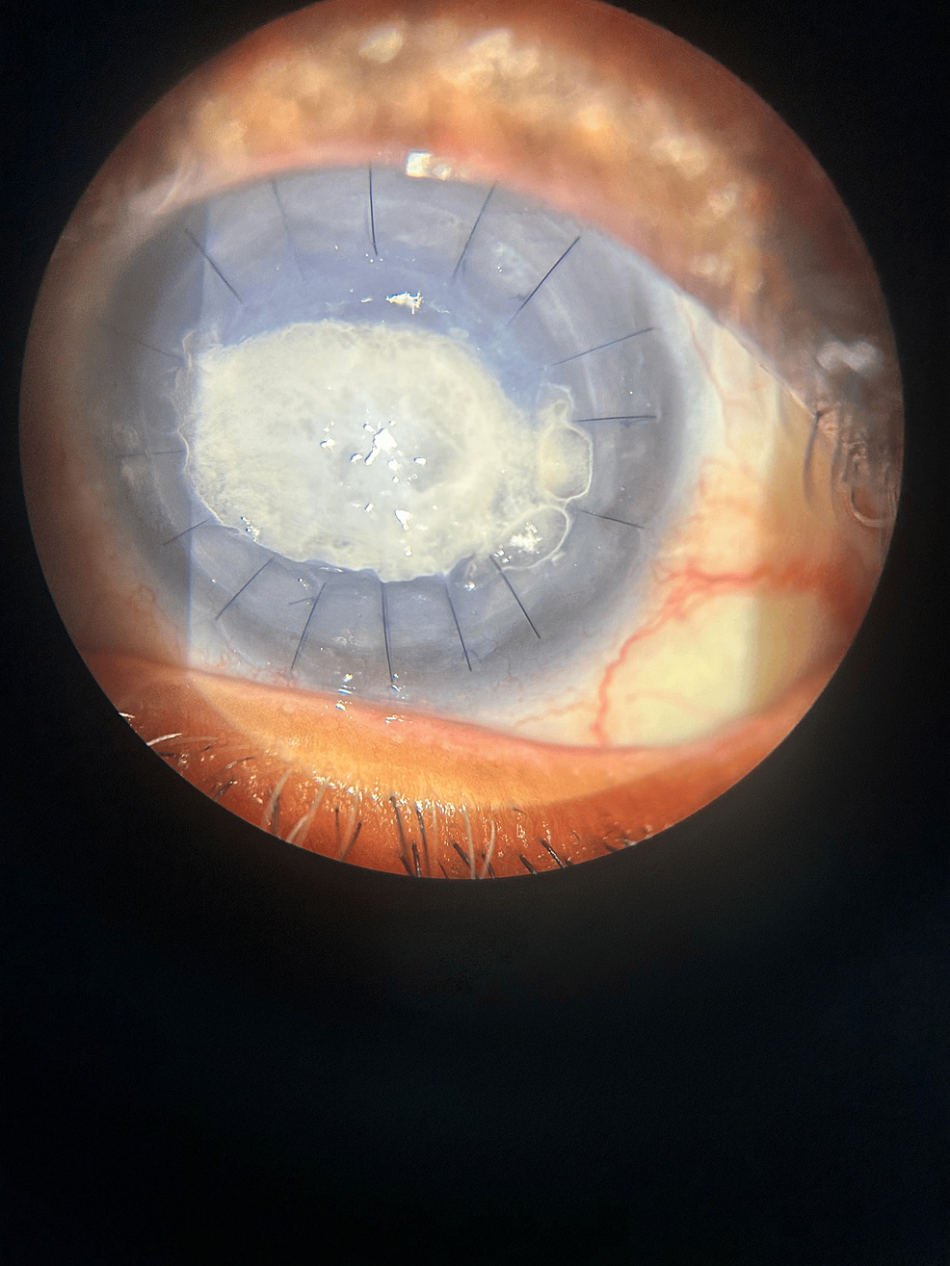
Anterior segment of the left eye showing crystalline deposits over the cornea, with 16 intact sutures and a healthy graft and suture site.

**Figure 2:**
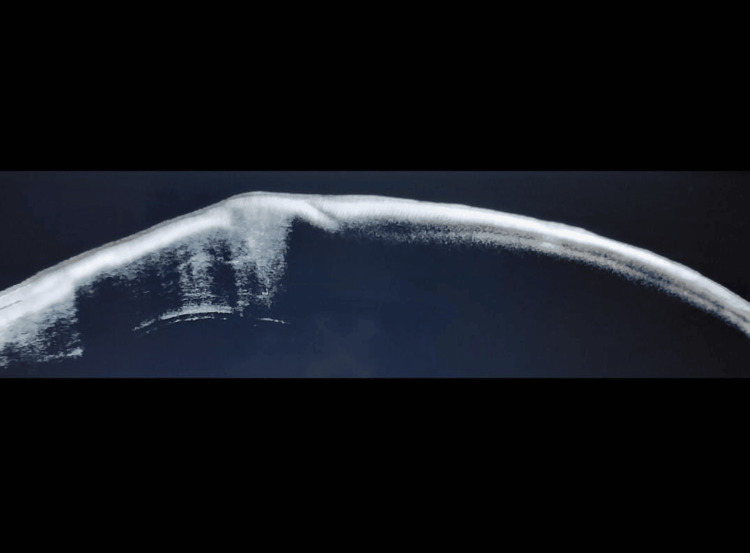
Anterior segment OCT of the left cornea showing corneal deposits involving the epithelium till the anterior stroma. OCT, Optical Coherence Tomography

**Figure 3:**
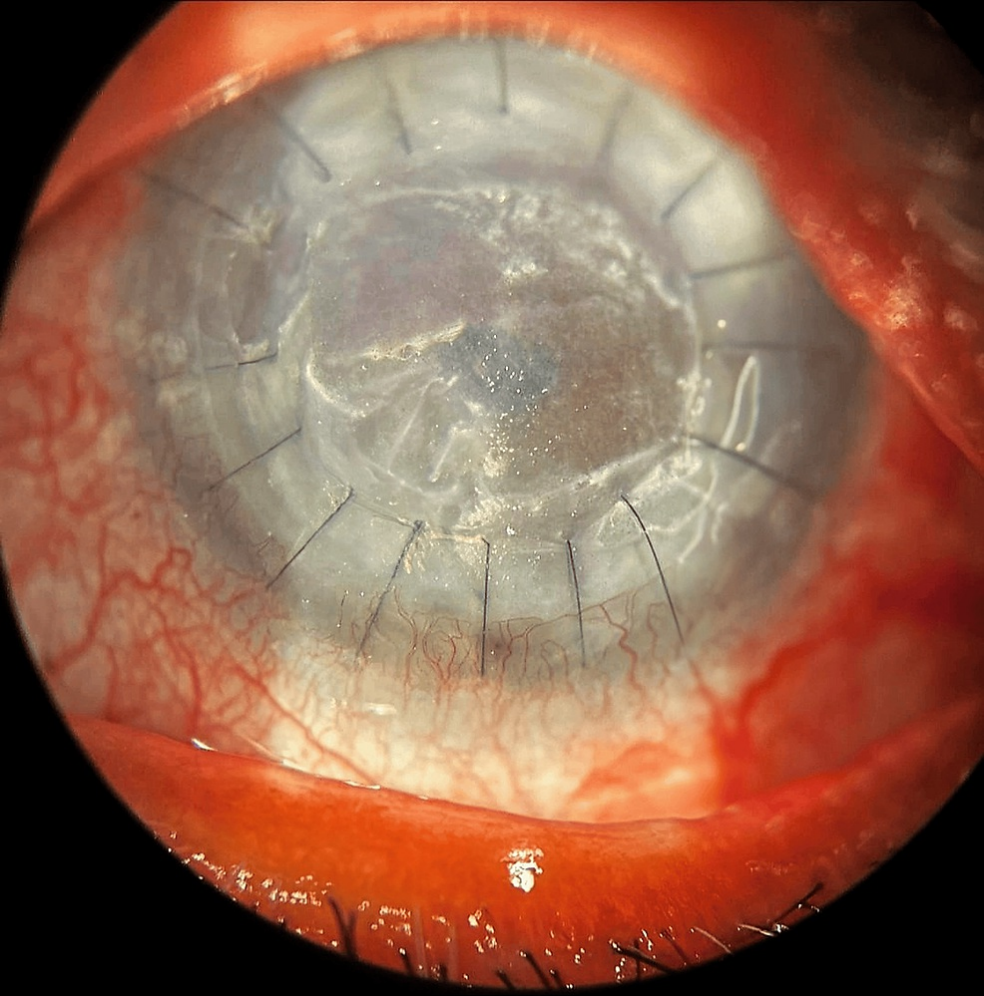
Cornea with clearing of the central area after scrapping of deposits with a 15-number blade.

**Figure 4:**
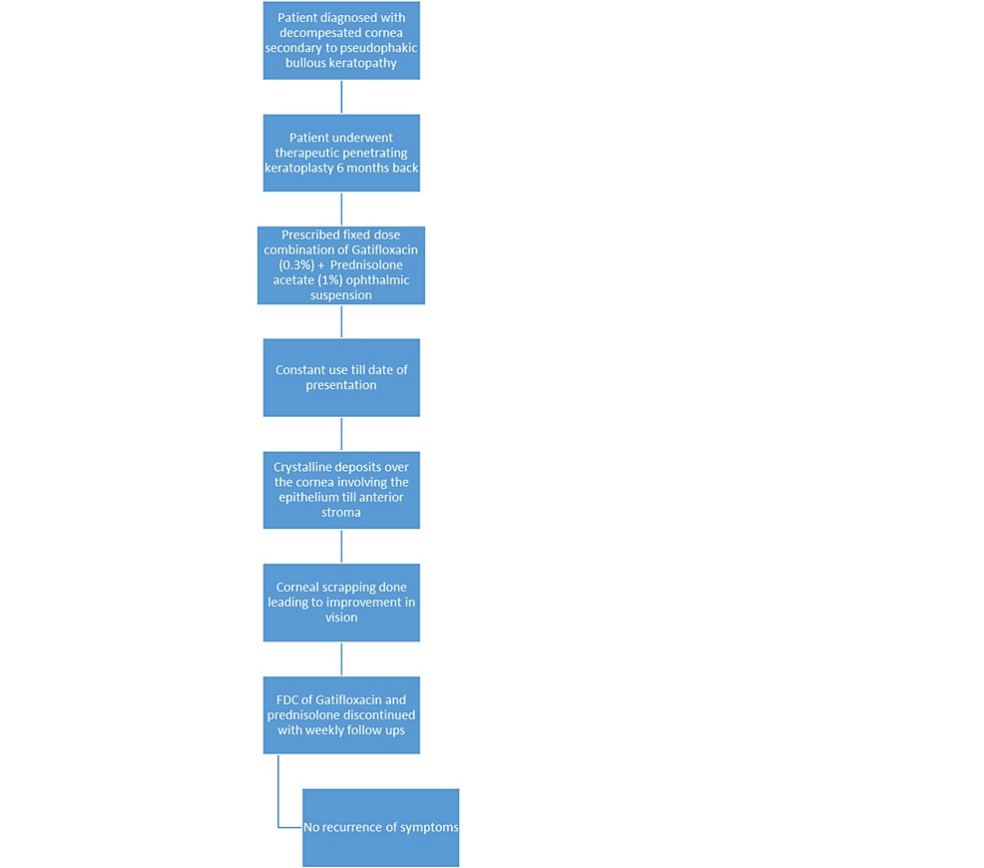
Sequence of surgical interventions, medications, and adverse events. Image credit: Chaitanya Modgil.

A 66-year-old female presented with a whitish patch on her left eye persisting for six months, accompanied by decreased vision in that eye. The patient was diagnosed with decompensated cornea secondary to Pseudophakic Bullous Keratopathy. She underwent penetrating keratoplasty six months ago. Following surgery, the patient has been using a fixed-dose combination of gatifloxacin (0.3%) and prednisolone acetate (1%) ophthalmic suspension eye drops continuously. The vision in her left eye was hand movements close to face with an accurate perception of rays, while her right eye had a vision of 6/9 with good pseudophakia. The anterior segment examination of her left eye revealed crystalline deposits over the cornea, with 16 intact sutures and a healthy graft and suture site (Figure 1). Anterior Segment Optical Coherence Tomography of the left cornea revealed cornea deposits involving the epithelium till the anterior stroma (Figure 2). The patient underwent corneal scrapping with a 15-number blade with a Bard-Parker handle under topical anesthesia (Figure 3). On Day 1 after scraping, there was a significant improvement in vision to 6/60. The fixed-dose combination of gatifloxacin and prednisolone was discontinued, and the patient was prescribed topical tobramycin eye drops four times a day, along with propylene glycol eye drops every two hours. Weekly follow-up was advised. There was no recurrence noted on subsequent follow-ups. Here is a schematic diagrammatic representation showing the chronology of the use of medication, adverse events, and surgery (Figure 4).

## Discussion

Topical fluoroquinolones are extensively utilized in ophthalmology due to their broad-spectrum antimicrobial activity, accessibility, and cost-effectiveness. Corneal deposition of these medications has been documented after their use in treating conditions such as bacterial keratitis, corneal transplant procedures, cataract surgery, and laser-assisted subepithelial keratectomy (LASEK) [2]. Common adverse effects following the use of fluoroquinolones include transient and mild dry eye, conjunctivitis, irritation, and watering. Corneal deposits following topical medication are frequently observed, typically affecting the corneal epithelium, although they can also manifest in the subepithelial or anterior stromal layers [3]. The most frequent adverse effect associated with the topical administration of fluoroquinolones is the development of a white crystalline precipitate or deposit on the surface of the cornea. This occurrence has been reported in as much as 17.6% of eyes treated with topical fluoroquinolones in human studies [4].

A study by Awwad et al. proposed that gatifloxacin, a fourth-generation fluoroquinolone, can precipitate intrastromal macroscopic crystalline deposits by compromising corneal epithelium. Tandem scanning corneal confocal microscopy verified the presence of crystals within the corneal tissue [5]. A case study on corneal crystalline deposits associated with topically applied gatifloxacin described a rare instance of stromal crystallization following topical treatment with gatifloxacin. Complete resolution of the corneal deposits occurred within 30 days after cessation of the drops, with no subsequent complications observed [6].

Huige et al. reported that when corticosteroids and beta-blocking agents are applied topically to a corneal wound or over sutures with impaired epithelium, it can lead to the development of calcific deposits in the superficial stromal layer of the cornea [7]. 

Upon discontinuation of the fixed-dose combination of gatifloxacin and prednisolone in this patient, symptoms did not reappear, indicating a probable causal relationship. Identifying these deposits early and discontinuing the medication promptly may lead to the resolution of the deposits.

## Conclusions

In summary, this case underscores the rare complication of corneal crystalline deposits due to topical gatifloxacin and prednisolone therapy following penetrating keratoplasty for pseudophakic bullous keratopathy. Management involved corneal scraping and modification of therapy, leading to the resolution of deposits, preservation of vision, and no future recurrence. Vigilant monitoring and cautious use of topical medications are crucial in preventing such adverse outcomes in ophthalmic patients.
